# The Use of a Matrix in Contouring Fillings

**Published:** 1890-10

**Authors:** H. A. Baker


### THE USE OF A MATRIX IN CONTOURING FILLINGS.[1]

BY DR. H. A. BAKER.

"After using all methods of separating by filing to get space between teeth for filling, I became fully convinced that the principle was entirely wrong, consequently gave the subject considerable thought." I was looking about for some other method to pursue when, in conversation with a professor in one of our dental colleges, I made to him the above statement. He replied, "If you come to my office some time, I think I can tell you of a method by which you will be able to save teeth." Upon availing myself of his invitation, I found his method was to bulge the fillings so that the tooth-substance would not come in contact, and was accomplished without previous separation.

After giving the subject further study, I came to the decision that it was an improvement at least, and went to work to put it to a practical test. Now, gentlemen, perhaps you can imagine my surprise when I found it a great deal easier to insert a contour filling in my mind than it was to put it in the distal surface of a twelfth-year molar. I persevered with the method, and the more I used it the more firmly was I convinced that the theory was correct.

About this time I began separating the teeth before filling,

---

[1] Read before the American Academy of Dental Science, Boston, Mass. April 2, 1890.

and with better results. While I was trying to simplify the work, it finally occurred to me, one day, that I had formerly used pieces of old files for a matrix in putting in a flat filling, which idea was, I believe, first introduced by Dr. Louis Jack. By drawing the temper and bending the file I had something approximating the contour of a natural tooth. Being more than pleased with the result, I felt that my future success, with careful work, was assured and much labor saved.

One day upon going to my case I accidentally took up a piece of thin copper. After bending this to the desired contour I inserted my wedge as I had formerly done when using pieces of file. The copper yielded to the pressure of the wedge, and if I had continued it, there would have resulted a flat filling. I removed the wedge and tied a ligature about the tooth and copper, by winding around the tooth from the cervical wall to the crown just as a thread would be wound around a spool. I then proceeded with my filling. After the filling was completed the copper was removed, and, to my surprise, I found that I had a gold filling of nearly the exact shape of the natural crown; the edges extended just a little beyond the periphery of the cavity and the surface appeared to be almost entirely polished. After burnishing, I found that I had very little finishing to do, and had a beautiful contour filling. As far as I know, this is the first time the ligated copper matrix was ever used for this purpose. Since that time the plain copper has been replaced by the silver-plated, which reflects light well and so illuminates the cavity. I now became very much encouraged, and grew very bold in making the cavities broader, with the idea of keeping the tooth-substance a greater distance apart. By this time I became fully convinced that I was in possession of the most satisfactory method of saving teeth by filling, and I have ever since been a strong advocate of contour filling. Moreover, I have brought the method before several dental societies, and I believe that the last paper that I read before this society was upon that subject. I was criticised after making the bold statement that I had never seen decay around a well-contoured gold filling in a proximal cavity.

I stand here to-night ready to repeat the same statement with one exception, and that relates to a patient's mouth where I have proved that gold is not a good filling, and that her teeth are of a very poor quality, and would be quite as liable to decay around a crown filling as about a proximal one. For some time past I have used nothing else but cement or copper amalgam for her teeth. I am also accused of making another bold statement,—that I have never seen Weston's cement dissolve at the cervical wall,—and I am also ready to renew this statement. While at that time I gave the credit to the cement, I will make the assertion that I have never seen any of the well-known cements decay at the cervical wall. I believe it is not the kind of cement but the method by which it is inserted. I wish it to be understood that the point in question refers to a proximal cavity.

You may smile when I give the whole credit to the matrix. My point is, that the matrix should be fitted closely around the tooth, and the filling inserted and forced in, producing a filling of uniform density. By using force it can be crowded in and overlap the periphery all around. The result is that the filling is as hard at the cervical wall as in any other part.

There are several kinds of matrices in use. It is difficult to decide between them; but this may be said, that the man who gave to the profession the steel matrix, encircling the whole or a part of the tooth and firmly held in position by some device, has given us one of the greatest boons that the profession is in possession of, and I do not hesitate to add—and I am ready to have it go upon the record—that any practitioner who does not avail himself of some of its forms now in use, and by its aid insert contour fillings in bicuspids and molars, reducing the operation to a simple filling, is far behind the times as an operator and does injustice to his patients by not giving them the most comfortable, serviceable, and conscientious work. The advantages of the matrix are too well known by the best and most careful operators to be discarded.

